# Competition between FLS and DFR regulates the distribution of flavonols and proanthocyanidins in *Rubus chingii* Hu

**DOI:** 10.3389/fpls.2023.1134993

**Published:** 2023-03-08

**Authors:** Ting Lei, Jun Huang, Haixiang Ruan, Wei Qian, Zhou Fang, Chunyang Gu, Niuniu Zhang, Yaxuan Liang, Ziyun Wang, Liping Gao, Yunsheng Wang

**Affiliations:** ^1^ School of Life Science, Anhui Agricultural University, Hefei Anhui, China; ^2^ State Key Laboratory of Tea Plant Biology and Utilization, Anhui Agricultural University, Hefei Anhui, China

**Keywords:** *Rubus chingii* Hu, flavonol synthase, dihydroflavonol 4-reductase, flavonols, proanthocyanidins

## Abstract

*Rubus chingii* Hu is a berry plant of the genus *Rubus* of the Rosaceae family, which has high nutritional and medicinal value and is rich in flavonoids. Flavonol synthase (FLS) and dihydroflavonol 4-reductase (DFR) compete for the common substrate dihydroflavonols to regulate the metabolic flux of flavonoids. However, the competition between FLS and DFR based on enzyme is rarely reported. Here, we isolated and identified two *FLS* genes (*RcFLS1* and *RcFLS2*) and one *DFR* gene (*RcDFR*) from *Rubus chingii* Hu. *RcFLSs* and *RcDFR* were highly expressed in stems, leaves, and flowers, although the flavonol accumulation in these organs was significantly higher than that of proanthocyanidins (PAs). The recombinant RcFLSs demonstrated bifunctional activities *via* hydroxylation and desaturation at the C-3α position having a lower Michaelis constant (Km) for dihydroflavonols than RcDFR. We also found that a low concentration of flavonols could significantly inhibit RcDFR activity. To investigate the competitive relationship between RcFLSs and RcDFR, we used a prokaryotic expression system (*E. coli*) to co-express these proteins. The transgenic cells expressing recombinant proteins were incubated with substrates, and the reaction products were analyzed. Furthermore, two transient expression systems (tobacco leaves and strawberry fruits) and a stable genetic system (*Arabidopsis thaliana*) were used to co-express these proteins *in vivo*. The results showed that RcFLS1 was dominant in the competition with RcDFR. Our results demonstrated that the competition between FLS and DFR regulated the metabolic flux distribution of flavonols and PAs, which will be of great significance for the molecular breeding of *Rubus* plants.

## Introduction

*Rubus chingii* Hu (Chinese raspberry) also known as “Fu-Pen-Zi” is a flavonoid-rich plant native to China. The ripe fruit of *R. chingii* is rich in nutrients and easy to digest; it can be eaten as berries or processed into jam. The immature fruit can be used as medicine after drying. Modern medicine has demonstrated that this herb could prevent frequent urination, relieve low back pain, improve eyesight, and prevent cancer (Zhang et al., 2015; Zeng et al., 2018; Yu et al., 2019). Secondary metabolites including flavonols, tannins, and PAs are abundant in *Rubus* plant and provide antioxidant and other health benefits (Moyer et al., 2002; Kaume et al., 2012).

Flavonoids, including flavones, flavonols, anthocyanins and PAs, are important secondary metabolites in dietary and medicinal plants (Wang et al., 2000). Flavonoids are found almost in all plant organs, including roots, stems, leaves, flowers, and fruits. They play important role in attracting pollinators (Winkel-Shirley, 2001; Saigo et al., 2020), protecting plants from UV irradiation (Li et al., 1993), regulating auxin transport (Peer et al., 2004), and promoting stamen development (Mo et al., 1992; Muhlemann et al., 2018). The flavonoid pathway is highly conserved across the plant kingdom. The biosynthesis of dihydroflavonols is identified as a critical branching point for the formation of downstream metabolites and is catalyzed by flavanone 3-hydroxylase (F3H). Flavonol synthase (FLS) and dihydroflavonol 4-reductase (DFR) then compete to catalyze the transformation of dihydroflavonols into flavonols and leucoanthocyanidins (co-precursors for PAs and anthocyanins synthesis), respectively. Thus, the expression levels of FLS and DFR are crucial for accumulating flavonols, PAs, and anthocyanins in plants (Tian et al., 2015; Luo et al., 2016; Qiu et al., 2022). Similarly, in grape hyacinth, competition between the FLS and the DFR may lead to eliminating blue pigmentation (Lou et al., 2014). The red-colored flower is successfully produced in tobacco by inhibiting the expression of the *FLS* gene while simultaneously overexpressing the gerbera *DFR* gene (Nakatsuka et al., 2007).

However, the accumulation of flavonoids is not always proportional to gene expression levels. For example, in some red apple varieties (*Malus domestica*), while the expression of *MdDFR* is higher than *MdFLS*, the anthocyanins content in its pericarp is much lower than flavonols (Takos et al., 2006). In the tea plant (*Camellia sinensis*), the expression of *CsFLSa* in buds is extremely high, but the buds mainly accumulate catechins (Jiang et al., 2020). In conclusion, FLS and DFR in plants regulate the downstream metabolic flux in the flavonoid pathway. This regulation is not only related to the expression level of key genes but also may influence by the catalytic ability of the enzymes. Revealing the competition between FLS and DFR from the perspective of enzymology will help to understand the dynamic equilibrium mechanism of flavonol and PAs in plants. However, most studies have focused on single functional or regulatory genes. The simultaneous co-expression of FLS and DFR to reveal the competitive relationship between FLS and DFR has not been reported. More research is needed to determine how to achieve the dynamic balance of flavonols and PAs.

In this study, *RcFLS*s (including *RcFLS1* and *RcFLS2*) and *RcDFR* were obtained from the genome of *R. chingii*, and their expression patterns were analyzed. The expression pattern of *RcFLS1* was consistent with the accumulation of flavonols, while *RcDFR* expression in fruits was consistent with PAs accumulation. However, *RcDFR* was expressed primarily in stems, flowers, and leaves, where the content of PAs was very low. Their enzymatic functions were verified by expression in *Escherichia coli* (*E. coli*) *in vitro*, and the kinetics showed that RcFLS1 has a higher affinity (Km) for dihydroflavonols than RcDFR. RcFLSs have also been shown to be bifunctional enzymes, exhibiting both flavonol synthase (FLS) and flavanone 3-hydroxylase (F3H) activities. We also found that flavonols were competitive inhibitors of RcDFR at low concentrations. In addition, RcFLSs and RcDFR could form an enzyme complex, according to protein-protein interaction assays. Using an *E. coli* prokaryotic expression system, two transient expression systems (tobacco leaves and strawberry fruits), and a stable genetic transformation system (*Arabidopsis thaliana*), we examined the competition between RcFLSs and RcDFR. The results showed that the competition between RcFLSs and RcDFR regulated the metabolic flux distribution of flavonols and PAs in *R. chingii*, in which RcFLS1 dominated the competition, and the metabolic flux was shifted to flavonols.

## Materials and methods

### Plant materials

Samples of different developmental stages and tissue parts of *R. chingii* plants were collected from Anhui agricultural university. All samples were collected separately during the growing and developmental seasons (May 2022). The tobacco (*Nicotiana benthamiana*) used in the experiments was grown in a climate chamber under a 16-h-light/8-h-dark cycle at 25 ± 1°C for 1 month before injection. *Arabidopsis thaliana* plants were grown in a climate chamber at 23 ± 1°C under a 16/8-h photoperiod.

### Extraction and quantitative analysis of polyphenols

Samples were pulverized into a fine powder in liquid nitrogen. Each 0.1 g sample was added with 2 mL extraction solution and extracted by low-temperature ultrasound for 30 min. The supernatant was collected after centrifugation at 13,000 rpm for 10 min at 4°C. The residues were extracted twice, and the supernatant was combined. Qualitative analysis of polyphenols using Q-TOF LC/MS was performed as previously reported (Wang et al., 2021).

### Recombinant proteins expression and purification

The pMAL-c2X vector (NEB, Beijing, China) was processed with the endonucleases BamHI and SalI (NEB, Beijing, China); the amplification of *RcFLS1*, *RcFLS2*, and *RcDFR* was performed using a primer set (**Table S1**) and then linked to the pMAL-c2X vector with MBP-Tag. The recombinant vectors were sequenced and transformed into *Escherichia coli* (*E. coli*) strain BL21 (DE3) (TransGen, Beijing, China). Positive strains were cultured in 50 mL Luria-Bertani (LB) medium with shaking at 37°C. When the optical density (OD) reached 0.8 at 600 nm, 80 μL of 1 M isopropyl β-d-1-thiogalactopyranoside (IPTG) was added to induce protein expression at 28°C for 8 h. The cells were re-suspended with Column Buffer (20 mM Tris-HCl, pH 7.4, 0.2 M NaCl, 1 mM EDTA) and disrupted through sonication for 30 min at 20% power on the ice. The RcFLS1, RcFLS2, and RcDFR recombinant proteins were purified through Amylose Resin (NEB, Beijing, China). The collected proteins were concentrated and detected by sodium dodecyl sulfate-polyacrylamide gel electrophoresis (SDS-PAGE).

### Enzymatic assays

For enzymatic reactions of RcFLSs, the reactions were performed in 100 mM Tris-HCl buffer (pH 7.5) containing 10 mM ascorbate, 10 μM FeSO_4_, 10 mM α-Ketoglutaric acid, 0.5 mg/mL catalase, 10 µg of the purified enzyme and 100 μM substrates in 100 μL of the mixture. The reactions were carried out at 30 °C for 30 min, and an equal volume of methanol was added to terminate the reaction. After that, the product was centrifuged to take the supernatant for High-Performance Liquid Chromatography (HPLC) analysis. HPLC was performed on an UltiMate3000 UPLC system (Thermo, MA, USA) connected to the HPH-C18 column (2.7 μm, 200 mm × 4.6 mm) at 30 °C and a flow rate of 0.4 mL/min. The mobile phase consisted of 100% acetonitrile (A) and 1.0% (v/v) acetic acid (B). The gradient profile was as follows: 0 min, 95% B/5% A; 2 min, 90% B/10% A; 4 min, 70% B/30% A; 6 min, 65% B/35% A; 14 min, 40% B/60% A; 16 min, 95% B/5% A; 18 min, 95% B/5% A. Flavanones and dihydroflavanols were analyzed at 287 nm, and flavonols were analyzed at 355 nm.

For enzymatic reactions of RcDFR, the reaction mixtures (100 μL) contained 0.1 M PBS buffer (pH 7.0), 1 mM NADPH, 10 µg of the purified enzyme, and 500 μM substrates. All the reactions were terminated after 1 h at 30 °C. Due to the instability of leucoanthocyanidins in the solution, an equal volume of butanol-hydrochloric acid (95:5, V/V) was added and reacted at 95°C for 30 min to form anthocyanidins (Petit et al., 2007). The content of anthocyanidins was determined by UV spectrophotometry at 530 nm.

### Kinetics analysis

For the RcFLSs recombinant protein, the following substrates were chosen to investigate the substrate specificity of the protein: Naringenin (N), Eriodictyol (E), Dihydrokaempferol (DHK) and Dihydroquercetin (DHQ). These substrate concentrations were set at 1 ~ 500 μM and reacted for 100 s ~ 300 s in the Tris-HCl buffer mentioned above. For the RcDFR recombinant protein, two kinds of dihydroflavonols (DHK and DHQ) were selected. These substrate concentrations were set at 5 ~ 800 μM and reacted for 1 h in the above reaction mixtures. Non-linear regression analysis of the velocity-concentration data fitted to the Michaelis–Menten was performed using GraphPad Prism 8 software to determine the maximal reaction rate (Vmax) and Michaelis constant (Km).

### Structural modeling and analysis

Discovery Studio 2019 (DS) (BIOVIA Corp, San Diego, CA, USA) was used to construct the structural model of RcDFR using the crystal structure of grape (*Vitis vinifera*) VvDFR (PDB DOI: 10.2210/pdb2C29/pdb) as a template. The molecular structures of ligands (dihydrokaempferol, dihydroquercetin, kaempferol and quercitrin) were retrieved from PubChem Compound (Wang et al., 2017). The CDOCKER program (Wu et al., 2003) available with the DS was utilized to dock ligands into a protein binding site. DS was also used to visualize protein-ligand complexes.

### Subcellular localization and protein interactions

For subcellular localization assays, full-length coding sequences (CDS) of the tested proteins were cloned into a pCAMBIA1305 vector carrying the green fluorescent protein (GFP) signal. *Agrobacterium* GV3101 carrying different vectors was infiltrated into tobacco leaves. After staining with 4’,6-diamidino-2-phenylindole (DAPI) for the nucleus, cells were visualized by the Olympus FV1000 confocal microscope (Olympus, Tokyo, Japan). For yeast two-hybrid (Y2H) assays, the full-length CDS of the tested proteins were cloned into pBT3-SUC and pPR3-N vectors and then co-transformed into yeast strain NMY51. Transformants were grown on SD-Trp-Leu medium and selected on SD-Trp-Leu-His-Ade medium at 30°C. For the collection of surface plasmon resonance (SPR) data, a Biacore T200 instrument and S senor chip CM5 (GE Healthcare, GA, USA) were used. Purified RcDFR-His protein was established on the sensor surface. Purified RcFLS1-His was injected in a concentration series of 1475 nM, 737.5 nM, 368.8 nM, 184.4 nM, and 92.2 nM RcFLS2-His was injected in a similar concentration series in another assay. Parameter settings and specific measurement methods were described previously (Peess et al., 2015). For the split-luciferase (LUC) complementation assays, pCAMBIA1300-nLUC and pCAMBIA1300-cLUC vectors were selected. Those *Agrobacterium* with constructed vectors were infiltrated into tobacco leaves. Luciferase activity was detected by Chemiluminescent Imaging System (Tanon, Shanghai, China) after growing in the climate chamber for 2 days.

### Expression of proteins in *E. coli* and enzyme activity assay

The genes were ligated to the pRSFDuet-1 vector in the same way described above. Positive BL21 (DE3) strains were screened and cultivated in 20 mL LB broth. After induction of protein expression in the presence of 0.8 mM IPTG at 28°C for 8 h, the substrates were added to 2 mL of culture at final concentrations of 100 μM. After incubation at 28°C for 8 h, 1 mL of culture was extracted with an equal volume of ethyl acetate. The ethyl acetate extracts were concentrated by vacuum centrifugal concentrator, and the residues were dissolved in 100 μL of chromatographic methanol for HPLC analysis. Another 1 mL of culture was sonicated and added with an equal volume of butanol-hydrochloric acid (95:5, V/V) at 95°C for 30 min. The supernatant was taken to determine anthocyanidin content by UV spectrophotometry at 530 nm. The anthocyanidin content was multiplied by ten in the final calculation because the flavonol concentration was concentrated by a factor of ten after extraction. Small samples of IPTG-induced bacterial cultures were analyzed with a western blot to confirm the levels of recombinant protein expression.

### Transient expression *in Vivo* by infecting tobacco and strawberry

The ORFs of *RcFLSs* and *RcDFR* were ligated to the vectors pCAMBIA1300-MYC and pCAMBIA1300-FLAG, respectively, in the same manner. Transfer 1 mL of *Agrobacterium* cultured overnight to 50 mL LB liquid medium and shake the culture at 28 °C. When the OD_600_ of the culture medium was approximately 0.8, the cells were resuspended in infection buffer (10 mM MgCl_2_, 200 μM acetosyringone, and 10 mM MES, pH 5.6). Fill the *Agrobacterium* suspension into a 1 mL syringe, and press the back of the syringe to inject the liquid from the lower epidermis of the leaf into the tobacco (CK was co-injected with pCAMBIA1300-MYC and pCAMBIA1300-FLAG; RcFLS1 was injected with pCAMBIA1300-RcFLS1-MYC; RcFLS2 was injected with pCAMBIA1300-RcFLS2-MYC; RcDFR was injected with pCAMBIA1300-RcDFR-FLAG; RcFLS1+DFR was co-injected with pCAMBIA1300-RcFLS1-MYC and pCAMBIA1300-RcDFR-FLAG; RcFLS2+DFR was co-injected with pCAMBIA1300-RcFLS2-MYC and pCAMBIA1300-RcDFR-FLAG). In the same way, the substrates (DHK and DHQ, 50μM) were supplemented to the injection site of tobacco leaves three days after infection. After the substrates were added for four hours, the leaves of the injection site were cut off, and the polyphenols were extracted in the same way described above. The relative quantification of polyphenols was performed by HPLC-triple quadrupole mass spectrometry (HPLC-QqQ-MS/MS) as previously reported (Jiang et al., 2018). The above extracts were pyrolyzed by adding 1/10 volume of concentrated hydrochloric acid at 95°C for 30 min, and HPLC detected the hydrolysates at 530 nm.

For strawberry (*Fragaria × ananassa* Duch. ‘Benihoppe’) injection, *Agrobacterium* cells were resuspended in infection buffer (10 mM MgCl_2_, 100 μM acetosyringone, and 10 mM MES, pH 5.6) as described previously (Zhao et al., 2019). The suspension was equably injected into the fruit from the center. Fruits were cultured at 23 ± 1°C, with a photoperiod of 16/8-h after *Agrobacterium* infection. Polyphenols were extracted three days after infection in the same manner as described above.

### Genetic transformation of *Arabidopsis* and determination of phenolic compounds

The recombinant pCAMBIA1300-RcFLS1/RcFLS2-MYC, pCAMBIA1300-RcDFR-FLAG, and empty vector were introduced into *Agrobacterium* GV3101 and were transformed into wild-type (Col-0) plants of *Arabidopsis thaliana*. The overexpressing plants were identified by semiquantitative PCR. Hybrid plants were obtained and verified with the overexpressing plant RcDFR as the female parent and RcFLS1 and RcFLS2 as the male parent. The extraction of polyphenols from *Arabidopsis* green pods was carried out in the same manner described above. The DMACA solution was prepared as described previously, and procyanidin content was determined at 640 nm (Payne et al., 2010). The total flavonoid content was determined using the aluminum chloride assay as previously reported (Karapandzova et al., 2015); here, the flavonoid content is regarded as flavonol.

### Immunoblot analysis

For *E. coli*, the induced-bacterial broth was diluted with distilled water to OD_600_ = 0.8. 1 mL of the diluted bacterial broth was centrifuged at 13,000 rpm and resuspended with 100 μL of distilled water. Add a suitable amount of resuspended solution to the SDS loading buffer, mix thoroughly, and boil for 5 min. The SDS-PAGE was used to separate the protein samples, and immunoblot analysis was performed using anti-HIS and anti-S tag monoclonal antibodies. For plant material, samples were taken 3 days after infection and ground into a powder at low temperature. An appropriate amount of the powder was transferred into 2 mL of pre-chilled extraction buffer (2 mM EDTA, 10% glycerol, 0.25% Triton X-100, 5 mM DTT, 100 μM PMSF, 150 mM NaCl, 50 mM Tris-HCl), mixed and incubated for 30 min on ice. The supernatant was collected as protein extract after centrifugation at 4°C, 13,000 rpm, for 10 min. The immunoblot analysis was performed using anti-MYC and anti-FLAG monoclonal antibodies.

## Results

### Gene expression and flavonoid content in different tissues of *Rubus chingii* Hu

*Rubus chingii* Hu, a member of the Rosaceae family, is mainly distributed in east China. Flavonols and PAs are the main active ingredients in *R. chingii*, and the key enzymes of the flavonoid pathway, FLS, and DFR, are essential for their synthesis. Based on the genome sequence and transcriptome information of *R. chingii*, all the homologs of *FLS* and *DFR* encoding genes (three *FLS* genes and two *DFR* genes in total) were screened (**Figure S1**). Based on their expression pattern, three candidate genes, *RcFLS1*, *RcFLS2*, and *RcDFR*, were successfully cloned. The FLS belonged to the 2-oxoglutarate-dependent dioxygenase (2-ODD) superfamily. Based on amino acid sequence similarity, the 2-ODD superfamily was classified into three classes: DOXA, DOXB, and DOXC (Kawai et al., 2014). Two different clades of the 2-ODDs were identified using phylogenetic analysis (**Figure S2**): one clade had FLSs and ANSs (DOXC47 clade), while the other contained F3Hs and FNSIs (DOXC28 clade). In addition, the FLS subclade could be divided into two clusters, with RcFLS1 belonging to cluster I and RcFLS2 belonging to cluster II, implying possible functional differences between RcFLS1 and RcFLS2. RcDFR belonged to the NADPH-dependent epimerase/dehydratase family, distinct from the 2-ODDs (**Figure S3**). RcFLS1 shared high homology with PpFLS of peach (*Prunus persica*), both of which belonged to the Rosaceae family, while RcDFR was the nearest to RhDFR of rose (*Rosa hybrid cultivar*), indicating evolutionary conservation in the Rosaceae family. We also investigated conserved amino acid residues and motifs important for FLS function (Chua et al., 2008) (**Figure S4**). By alignment with other sequences of 2-ODDs involved in flavonoid biosynthesis, it is discovered that RcFLSs possess important motifs of “PxxxIRxxxEQP,” “CPxPxLAL” and “SxxTxLVP.” All sequences shared motifs unique to 2-ODDs involved in the binding of ferrous iron (green arrows) and 2-oxoglutarate (blue arrows).

**Figure 1:**
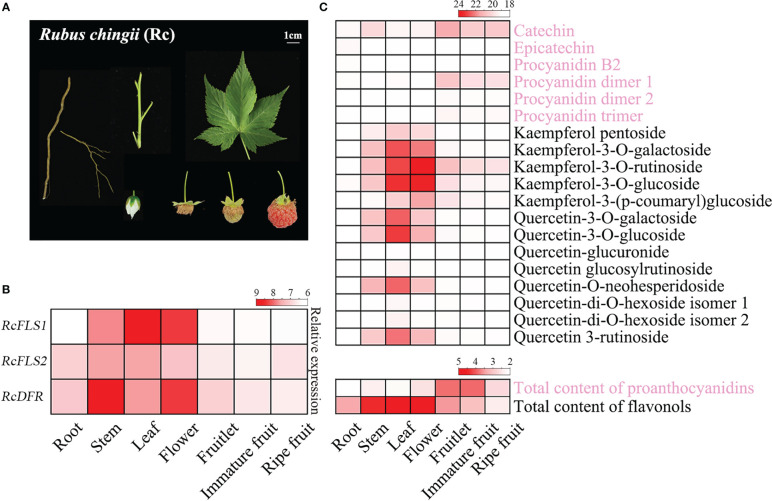
Expression profiles of genes and accumulation patterns of flavonoids in *R. chingii*. **(A)** Different tissues of *R. chingii* were used in this experiment, including root, stem, leaf, flower, fruitlet, immature fruit, and ripe fruit. **(B)** The expression profiles of *RcFLSs* and *RcDFR* in different tissues of *R. chingii*. **(C)** Heat map of PAs and flavonols accumulation in different tissues of *R. chingii*. A total of 19 flavonoids, including 6 procyanidins and 13 flavonols, were identified and quantified using Q-TOF LC/MS (up); The contents of total PAs and flavonols were determined by colorimetric methods (down).

Transcriptome and polyphenol metabolome analyses were performed to clarify the relationship between gene expression and metabolite accumulation (**Figure 1**). The expression levels of *RcFLSs* and *RcDFR* in different tissues and different developmental stages of fruit were analyzed (**Figure 1B**). The results revealed that *RcFLS1* had a high expression level in the leaves and flowers, followed by the stems, and a low expression level in the roots and all fruit stages (**Figure 1B**). The expression level of *RcFLS2* was lower in the leaves, flowers, and stems than *RcFLS1* and higher in the roots and all fruit stages. *RcDFR* was highly expressed in stems and flowers and moderately expressed in all fruit stages, and the expression level gradually decreased with fruit ripening.

In addition, 19 flavonoids (including 6 proanthocyanidins and 13 flavonols) were identified and quantified by Q-TOF LC/MS, and the contents of total PAs and flavonol were determined by colorimetric methods (**Figure 1C**). The highest PAs content was found in immature fruit and fruitlet (11.60 mg/g and 11.25 mg/g, respectively), while the highest flavonol content was found in the leaf and flower of *R. chingii* (21.57 mg/g and 20.82 mg/g, respectively). In addition, catechin and kaempferol-3-O-rutinoside were the most abundant PAs and flavonols in *R. chingii*, respectively. The polyphenol metabolome analysis indicated anthocyanins were generally not found in *R. chingii* [Carotenoids have been reported to impart the reddish color to ripe fruit (Li et al., 2021)], and only several PAs (including (+)-catechin and its procyanidin dimer) were detected (**Figure 1C**). PAs mainly accumulated in each fruit stage, and the content gradually decreased with fruit development. Meanwhile, flavonols are primarily accumulated in the leaves and flowers, and only kaempferol derivatives were a little accumulated in each fruit stage (**Figure 1C**).

The expression pattern of *RcFLS1*, but not *RcFLS2*, was consistent with the concentration of flavonols in *R. chingii*, suggesting *RcFLS2* might not be a major gene determining flavonols accumulation (**Figures 1B, C**). The transcripts of *RcDFR* gradually decreased with fruit development, which was consistent with the concentration of PAs and indicated the involvement of this gene in PAs biosynthesis. Interestingly, the expressions of *RcDFR* in the stem and flower were significantly higher than in all fruit stages, where PA accumulation was low (**Figures 1B, C**). The contradiction between the level of *RcDFR* expression (high expression in the stem) and PA accumulation (high accumulation in the fruitlet) requires further investigation.

### Analysis of the enzyme characteristics of RcFLSs and RcDFR recombinant proteins

**Figure 2:**
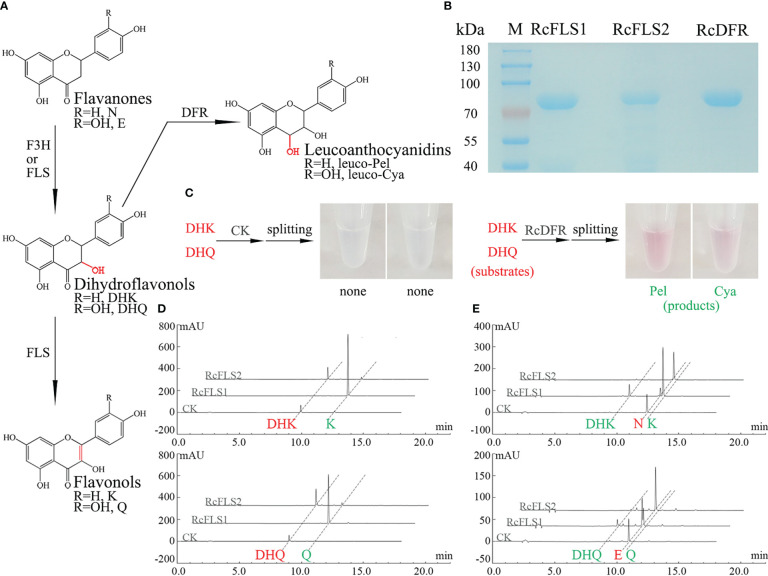
Analysis of the recombinant RcFLSs and RcDFR enzymatic reaction products. **(A)** Flavonoid biosynthesis pathway leading to the synthesis of flavonols and leucoanthocyanins. N, Naringenin; E, Eriodictyol; DHK, Dihydrokaempferol; DHQ, Dihydroquercetin; K, Kaempferol; Q, Quercetin. leuco-Pel, Leucopelargonidin; leuco-Cya, Leucocyanidin. **(B)** SDS-PAGE analysis of the purified recombinant fusion proteins **(C)** Analysis of the enzyme reactions with the recombinant RcDFR fusion proteins *in vitro*. **(D, E)** Analysis of the reaction products obtained from the recombinant RcFLSs fusion proteins *in vitro*, using dihydroflavonols and flavanones as substrates, respectively. The enzyme reaction substrates are shown in red, and the products are shown in green.

Several pMAL-c2X vectors carrying different genes were constructed and effectively expressed in *E. coli* BL21 (DE3) to investigate the enzymatic properties of RcFLSs and RcDFR. An SDS-PAGE electrophoretogram revealed distinct target bands obtained after induction with IPTG and purification using amylose resin (**Figure 2B**).

We detected anthocyanidin products that split from leucoanthocyanidins under high temperatures and acidic conditions because the leucoanthocyanidin produced by the RcDFR enzyme is highly unstable and difficult to see directly by HPLC. Here, the enzyme reaction substrates are shown in red, and the products are shown in green. The pink products were observed in the reaction of the recombinant protein using different substrates, dihydrokaempferol (DHK) and dihydroquercetin (DHQ) (**Figure 2C**). HPLC analysis showed that both RcFLS1 and RcFLS2 could catalyze dihydroflavonols (DHK and DHQ) to form their corresponding flavonols (Kaempferol, K and Quercetin, Q), while RcFLS1 had a higher catalytic capacity (**Figure 2D**). In addition, when flavanones (Naringenin, N and Eriodictyol, E) were fed as substrates, dihydroflavonols (DHK and DHQ) and flavonols (K and Q) were produced from RcFLS1, while RcFLS2 performed less active and produced only a few of dihydroflavonols (**Figure 2E**). It was suggested that RcFLSs were bifunctional enzymes with both hydroxylation and desaturation at the C-3α position.

Enzyme kinetic analysis was carried out to investigate further the affinity of RcFLSs and RcDFR with different substrates. The enzyme kinetics of RcFLS2 could not be determined accurately because of its low activity. The RcFLS1 and RcDFR enzyme catalysis rate curves, which were dependent on substrate concentration, fit the Michaelis–Menten equation (**Table 1** and **Figure S5**). RcFLS1 displayed a similar affinity (Km) to the different substrates, but the catalytic efficiency (Kcat/Km) was the highest for dihydroflavonols. It was discovered that the most suitable substrate for RcFLS1 was dihydroflavonols rather than flavanones. RcDFR was found to have a higher affinity (Km) and higher enzyme catalytic efficiency (Kcat/Km) for DHK than DHQ, indicating that DHK was the most suitable substrate for RcDFR. In addition, RcFLS1 displayed higher affinity (the Km values for DHK and DHQ were 33.94 and 56.93 μM, respectively) and catalytic efficiency (the Kcat/Km values for DHK and DHQ were 2206.83 and 2616.67 s^-1^·M^-1^, respectively) for the common substrates dihydroflavonols, compared to RcDFR (the Km values for DHK and DHQ were 47.24 and 124.93 μM, and the Kcat/Km values for DHK and DHQ were 1704.07 and 845.33 s^-1^·M^-1^, respectively).

**Table 1:** Kinetic parameters of recombinant RcFLS1 and RcDFR proteins.

Enzyme	Substrate	Vmax	Km	Kcat	Kcat/Km
		(pKat*mg^-1^)	(μM)	(ms^-1^)	(s^-1^ M^-1^)
**RcFLS1**	N	56.40 ± 8.11	43.60 ± 14.59	4.52 ± 0.65	103.55
	E	179.07 ± 3.78	34.51 ± 9.34	14.33 ± 0.30	415.43
	DHK	935.70 ± 78.70	33.94 ± 8.66	74.90 ± 6.30	2206.83
	DHQ	1861.00 ± 233.36	56.93 ± 16.36	148.97 ± 18.68	2616.67
**RcDFR**	DHK	997.87 ± 31.27	47.24 ± 5.79	80.51 ± 2.52	1704.07
	DHQ	1309.00± 35.16	124.93 ± 11.41	105.61 ± 2.84	845.33

Values were means ± SD of 3 biological replications.

### Flavonols significantly inhibited the activity of RcDFR

**Figure 3:**
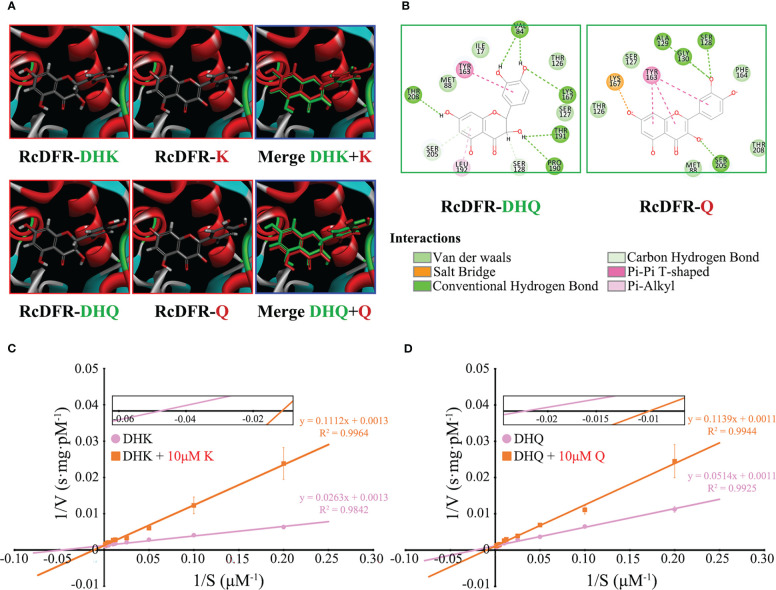
Low concentrations of flavonols competitively inhibited the activity of RcDFR. **(A)** Homology models of RcDFR with docked dihydroflavonols (DHK, DHQ) and flavonols (K, Q) ligands (partially enlarged view). The protein is shown in ribbon presentation (α-helices: red, β-sheets: blue, turns: green). DHK, Dihydrokaempferol; DHQ, Dihydroquercetin; K, Kaempferol; Q, Quercetin. **(B)** 2D interaction pattern of RcDFR-DHQ and RcDFR-Q. Key amino acid residues involved in docking were shown. **(C)** The effect of 10 μM K on the Vmax and Km of RcDFR when the substrate was DHK. **(D)** The effect of 10 μM Q on the Vmax and Km of RcDFR when the substrate was DHQ. The X-intercept was magnified to facilitate comparison. The pink circle and orange rectangle indicate that the substrate was dihydroflavonols and dihydroflavonols + 10 μM flavonols, respectively.

To investigate whether flavonols inhibit the activity of RcDFR, we first constructed a homologous structural model. Since the amino acid sequence similarity between RcDFR and VvDFR was about 80%, the protein crystal model of VvDFR was used as a template. After that, four flavonoid molecules (including DHK, DHQ, K, and Q) were used to perform precise molecular docking with RcDFR (**Figure 3A**). The docking results showed that DHK or DHQ and K or Q occupied the same catalytic space and shared similar key amino acid residues like TYR163 and LYS167 (**Figures 3A, B**). PHE164 has been reported to play an important role in regulating DFR by flavonols (Aiguo et al., 2022). RcDFR contained this key amino acid residue, which influenced protein binding to flavonol ligands *via* van der Waals forces (**Figure 3B**). In addition, we investigated the effects of K or Q at a low concentration (10 μM) on the Vmax and Km of RcDFR to better understand the mechanism by which flavonols inhibit RcDFR. According to the Lineweaver-Burk plot, the Y-intercept in the presence of 0 or 10 μM flavonol was virtually the same, whereas the X-intercept increased in the presence of 10 μM flavonol, which was consistent with the typical plot of competitive inhibitors (**Figures 3C, D**). These data indicated that flavonols were competitive inhibitors of RcDFR at low concentrations. Therefore, product inhibition affected the competition between FLS and DFR, making FLS more dominant and thereby regulating metabolic flux.

### Subcellular localization and protein interaction

**Figure 4:**
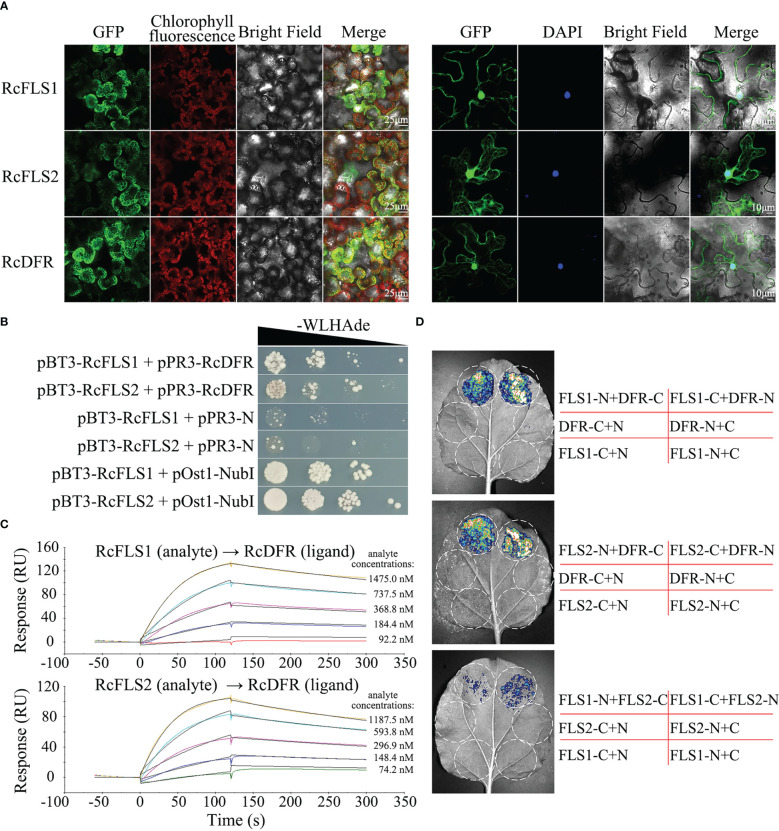
Subcellular localization and protein-protein interaction of RcFLSs and RcDFR. **(A)** Subcellular localization of RcFLSs and RcDFR enzymes as determined by the transient expression of their fluorescence-protein chimeras in tobacco leaf cells. DAPI was used to label nuclear DNA. Mesophyll cells (left) and epidermal cells (right). **(B)** Yeast two-hybrid assays of the interactions between RcFLSs and RcDFR. The yeast was cultured on an appropriate selection medium with cells harboring pOst1-NubI as a positive control and pPR3-N as a negative control. -WLHAde indicates that tryptophan, leucine, histidine, and adenine are missing on the SD medium. **(C)** Surface plasmon resonance (SPR) assays of the binding behavior between RcFLSs and RcDFR *in vitro*. Purified RcDFR protein was established as a ligand on the sensor surface. Five concentrations of each protein (RcFLSs) were tested as analytes. **(D)** Split-luciferase (LUC) complementation assays of the interactions between RcFLSs and RcDFR *in vivo*. N, nLUC; C, cLUC.

The subcellular localization of RcFLSs and RcDFR was examined by analyzing the intracellular localization patterns of fluorescence from their GFP-chimeric proteins in tobacco cells. The nucleus was visualized using the mounting medium containing 4’,6-diamidino-2-phenylindole (DAPI) (**Figure 4A**). The results showed similar localization patterns to RcFLSs and RcDFR, such as cytoplasmic space and nucleus. To further verify the interactions between RcFLSs and RcDFR, yeast two-hybrid surface plasmon resonance (SPR) and split-LUC assays were performed, respectively. For yeast two-hybrid assays, co-transformants (named on the left) were selected on SD-Trp-Leu-His-Ade medium in a dilution series of 1, 10^-1^, 10^-2,^ and 10^-3^ (**Figure 4B**). The growth of yeast cells on the selective medium (lines 1 and 2) showed that RcFLSs interacted with RcDFR. In addition, the interactions between RcFLSs and RcDFR in yeast two-hybrid assays were relatively weak compared to the positive control. SPR has been widely used in biomolecular interaction analysis. To confirm and extend these results, we turned to an *in vitro* approach and used SPR to analyze the interaction between RcFLSs and RcDFR (**Figure 4C**). We first established a purified His-RcDFR protein on the sensor surface. The purified His-RcFLS1 protein was injected with a concentration gradient from low to high, while the purified His-RcFLS2 protein was injected with a similar concentration series. The results showed that the response value (RU) raised with the increase of RcFLSs protein concentration, indicating an interaction between RcFLSs and RcDFR. We used split-LUC complementation assays to confirm this interaction *in vivo* (**Figure 4D**). The *Agrobacterium* with constructed vectors was first infiltrated into tobacco leaves. The results showed that the co-transformation of RcFLSs and RcDFR had significantly stronger luciferase activities than the control, while the interaction between RcFLS1 and RcFLS2 was generally weak. The results indicated that RcFLSs and RcDFR targeted the same subcellular localization to interact with appropriate partners and might form functional complexes in metabolic processes.

### Competition between RcFLSs and RcDFR in *E. coli*

**Figure 5:**
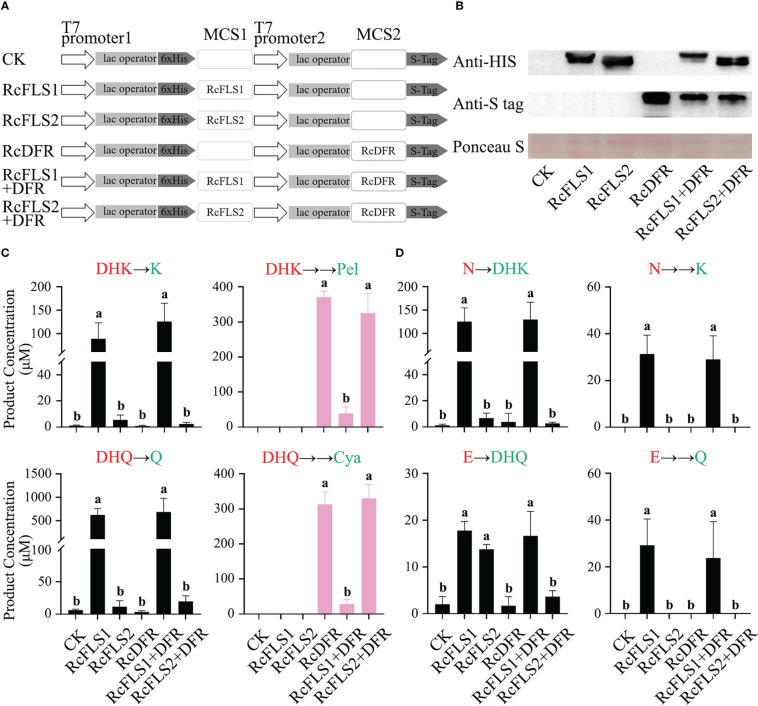
Assays of competition between RcFLSs and RcDFR in *E*. *coli*. **(A)** pRSFDuet-1 plasmid construct used for the expression of RcFLSs and RcDFR. MCS, multiple cloning sites. **(B)** Immunoblot assays for the recombinant RcFLSs-HIS and RcDFR-S tag fusion proteins. Ponceau S staining for total protein normalization. **(C, D)** The levels of flavonoid products from each reaction were determined using dihydroflavonols and flavanones as substrates, respectively. Different substrates (red) were fed, and the corresponding products (green) were detected. Error bars indicate ± SD from three repeats. Statistical significance was determined by one-way ANOVA with *post hoc* Tukey’s tests (P < 0.05) and indicated by letters.

To explore the relationship between *RcFLSs* and *RcDFR* in terms of substrate utilization, these genes were cloned into the pRSFDuet-1 vector (**Figure 5A**) and transformed into *E. coli* BL21 (DE3). The expression of fusion proteins was successfully induced by IPTG in the bacterial expression system and examined *via* immunoblot analysis (**Figure 5B**).

Transgenic cells expressing recombinant proteins were incubated with substrates (dihydroflavonols and flavanones), and the reaction products were analyzed (**Figures 5C, D**). When the substrate was dihydroflavonols, we examined the product of FLS (flavonols) and the product of DFR (anthocyanins produced by pyrolysis), respectively. When the substrate was flavanones, we detected the products of the two-step reaction of FLS (dihydroflavonols and flavonols).

Consistent with the *in vitro* enzymatic activity results, RcFLSs exhibited both F3H and FLS activity, while RcFLS1 had higher enzymatic activity than RcFLS2 (**Figure 5C**). Moreover, when RcFLS1 and RcDFR were co-expressed in *E. coli*, it was discovered that the production of anthocyanidins was significantly reduced compared to RcDFR expression alone. Still, the production of flavonols was maintained at a high level and was not significantly different from RcFLS1 expression alone (**Figure 5C**). When RcFLS2 and RcDFR were co-expressed simultaneously in *E. coli*, we found no significant changes in the production of flavonols and anthocyanidins compared to when RCFLS2 and RCDFR were expressed separately (**Figure 5C**). When the substrate was flavanones, RcFLS1 catalyzed it to dihydroflavonols and flavonols, and the yield was still not significantly different from that of RcFLS1 and RcDFR co-expression (**Figure 5D**). However, when RcFLS2 and RcDFR were co-expressed, the production of dihydroflavonols, particularly DHQ, was significantly reduced compared to RcFLS2 expression alone (**Figure 5D**). Therefore, RcFLS1 exhibited extreme competitiveness, directing substrate flow to flavonols, while RcDFR had moderate competitiveness.

### Competition between RcFLSs and RcDFR in tobacco leaves

**Figure 6:**
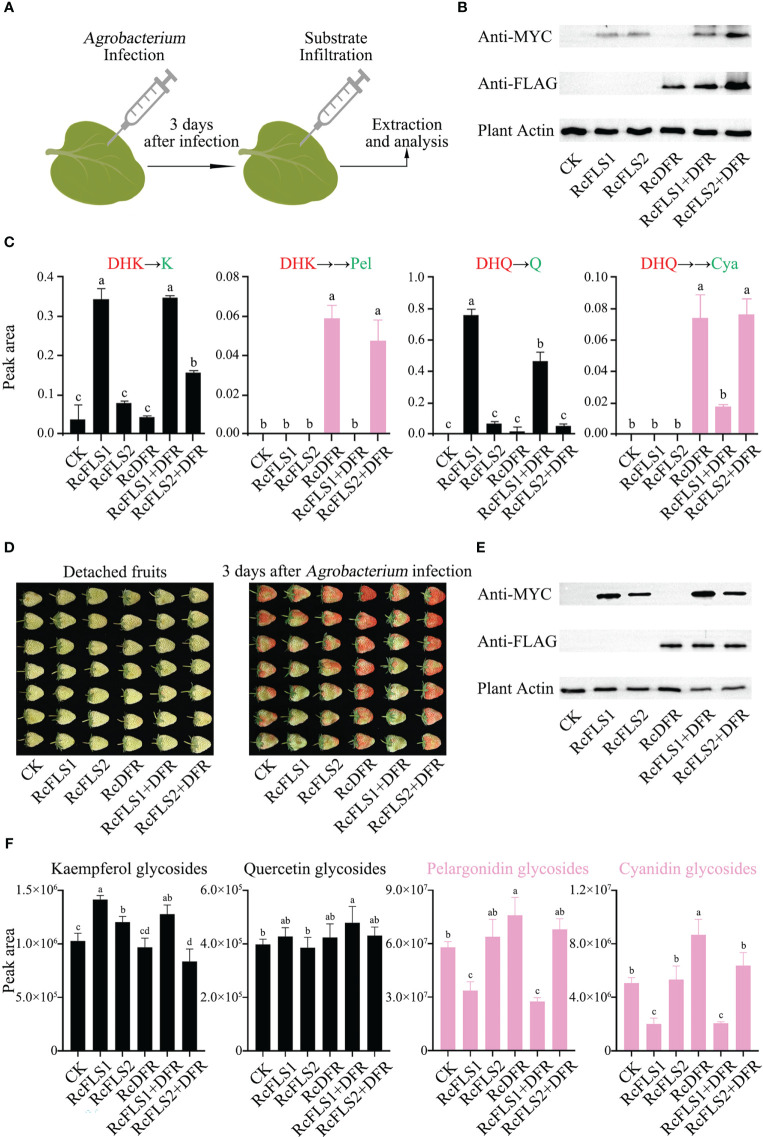
Assays of competition between RcFLSs and RcDFR in tobacco leaves and strawberry fruits. **(A)** *Agrobacterium*-mediated transient transformation and reaction system in tobacco leaves. **(B)** Immunoblot assays for the recombinant RcFLSs-MYC and RcDFR-FLAG fusion proteins in tobacco leaves. Plant-actin was used as a loading control. **(C)** Relative contents of flavonols and anthocyanins in pyrolysis products from tobacco leaves after co-injection of substrates. The substrates (red) are DHK (left) and DHQ (right). **(D)** Phenotypic changes of detached strawberry fruits infected with *Agrobacterium* for three days. **(E)** Immunoblot assays for the recombinant RcFLSs-MYC and RcDFR-FLAG fusion proteins in strawberry fruits. **(F)** Relative accumulation of flavonol glycosides and anthocyanins extracted from transiently infected fruits. Error bars indicate ± SD from three repeats. Statistical significance was determined by one-way ANOVA with *post hoc* Tukey’s tests (P < 0.05) and indicated by letters.

A transient expression system in tobacco leaves was established to investigate the relationship between RcFLSs and RcDFR *in vivo* (**Figure 6A**). 3 days after infection with *Agrobacterium*, the substrates (DHK and DHQ) were supplemented to the injection site of tobacco leaves for reaction, and then samples were taken for detection. At the same time, immunoblot analysis was used to examine the protein expression in tobacco leaves (**Figure 6B**). The amount of flavonol derivatives in transiently overexpressed RcFLS1 and RcFLS1+DFR tobacco leaves was significantly higher than that in control, but there was no significant difference between them (**Table S2**). The results showed that the expression of RcDFR did not inhibit the synthesis of flavonols in tobacco leaves, which was consistent with the results of feeding experiments in *E. coli* (**Figure 5C**).

Furthermore, we split the tobacco leaf extracts under high temperatures and acidic conditions, and the pyrolysis products were detected using HPLC (**Figure 6C**). When DHK was co-injected, we found that K formed in the pyrolysis products, and the yield in the RcFLS1 and RcFLS1+DFR tobacco leaves was much higher than that of the other tobacco leaves, indicating that RcFLS1 was dominant in the competition with RcDFR (**Figure 6C**). Similarly, When DHQ was co-injected, RcFLS1 tobacco leaves exhibited significantly higher Q contents than other tobacco leaves in the pyrolysis products. In addition, the yield of flavonols in tobacco leaves of RcFLS2 was slightly higher than that of the control, but the difference was not significant. Strangely, when the substrate was dihydrokaempferol, the kaempferol content in RcFLS2+RcDFR tobacco leaves was significantly higher than that in RcFLS2, which requires further study. We also discovered two anthocyanidin monomers linked to RcDFR activity in the pyrolysis products (**Figure 6C**). While there was no significant difference between RcFLS2+DFR and RcDFR tobacco leaves, the anthocyanidin content of pyrolysis products in RcFLS1+DFR tobacco leaves was significantly lower than that in RcDFR tobacco leaves. Interestingly, we found that the amount of Q in the pyrolysis products of RcFLS1+DFR tobacco leaves was substantially lower than that of RcFLS1, which may be due to the complexity of *in vivo* experiments in plants. The findings demonstrated that RcFLS1 dominated the competition, leading to a greater substrate flow to flavonols, whereas RcFLS2 had the weakest competitiveness.

### Competition between RcFLSs and RcDFR in strawberry fruits

To demonstrate the accumulation of anthocyanidins more visually, we utilized a transient expression system in strawberry fruits. After inoculating with *Agrobacterium*, for 3 days the phenotypic changes in the fruits were observed (**Figure 6D**), and immunoblot analysis was used to examine the protein expression in strawberry detached fruits simultaneously (**Figure 6E**). The strawberry fruits of RcFLS1 and RcFLS1+DFR were generally cyan, while RcDFR and RcFLS2+DFR fruits were red, and RcFLS2 fruits were similar to the control (**Figure 6D**).

Furthermore, we performed relative quantification for each class of compounds (kaempferol, quercetin, pelargonidin and cyanidin glycosides), and the results showed that the content of flavonoids in strawberry fruits was different (**Figure 6F**). The monohydroxy compounds, including kaempferol and pelargonidin glycosides, were mainly accumulated in strawberry fruits. The content of kaempferol glycosides in RcFLS1, RcFLS2, and RcFLS1+DFR strawberry fruits was significantly increased than that in control, while that in RcFLS2+DFR strawberry fruits was significantly decreased. In contrast, anthocyanins (including pelargonidin and cyanidin glycosides) were significantly increased in RcDFR strawberry fruits but decreased in RcFLS1 and RcFLS1+DFR strawberry fruits. In addition, the content of quercetin glycosides was significantly higher in RcFLS1+DFR strawberry fruits than in the control fruits, while the content in other strawberry fruits had no significant difference, which could be related to the substrate preferences of FLS and DFR. Finally, the RcFLS1 protein, transiently expressed in strawberry fruits, demonstrated extreme competitiveness, directing metabolic flux toward flavonols. At the same time, the RcFLS2 protein also had a certain catalytic ability but was not dominant in the competition.

### Competition between RcFLSs and RcDFR in *Arabidopsis thaliana*

To further investigate the relationship between RcFLSs and RcDFR *in vivo*, we used *agrobacterium*-mediated transformation to obtain stable RcFLS1, RcFLS2, and RcDFR *Arabidopsis* and crossed to generate co-expression plants RcFLS1+DFR and RcFLS2+DFR (**Figure S6**). The phenotypes of these plants were similar, but the content of polyphenols in their green pods was significantly different. RcFLS1 and RcFLS1+DFR overexpression plants showed the highest flavonol but lowest PA content (**Figure S6**), indicating that when RcFLS1 and RcDFR were co-expressed, the common substrate in flavonoid metabolism was primarily consumed by RcFLS1, with the product flowed to flavonols. The flavonol content of RcFLS2 overexpression plants increased but not significantly compared to the control, which was related to the lower flavonol synthesis activity of RcFLS2. Simultaneously, the PA content of RcFLS2+DFR and RcDFR overexpression plants was significantly higher than the other plants (**Figure S6**), suggesting that when RcFLS2 and RcDFR were co-expressed, the product flowed primarily to PAs.

## Discussion

*Rubus chingii* Hu is a characteristic traditional Chinese medicinal (TCM) plant. The immature fruit has wide medicinal and dietary values because of its unique pharmacological effects, and consumers appreciate the ripe fruit for its unique flavor and nutritional value. According to current findings, *R. chingii* has been reported to contain abundant healthy chemical constituents, including flavonoids, terpenoids, and hydrolytictannins. The main flavonoids in *R. chingii* were kaempferol, quercetin, and their derivatives (Yu et al., 2019). We found that flavonol glycosides were highly accumulated in leaves, flowers, and young stems of *R. chingii*, while a certain amount of PAs was accumulated in fruits at different development stages. The key genes of flavonoid synthesis in *R. chingii* have not been reported. In this study, two *RcFLS* and one *RcDFR* gene were isolated from the genome of *Rubus chingii* Hu. The role of these genes in the accumulation of flavonoids in *R. chingii* needs to be further explored.

### RcFLSs are bifunctional enzymes that exhibit F3H and FLS activity

Flavonoids have rich health functionality, including anticancer, antioxidant, antibacterial and prevention of cardiovascular diseases (Sefton et al., 1990; Finger et al., 1991; Shen, 2022). However, mechanisms involved in the biosynthesis of flavonoids remain unclear. In the flavonoid biosynthesis pathway, F3H and FLS catalyze hydroxylation and desaturation reaction of flavanones, which belong to the 2-oxoglutarate-dependent dioxygenase (2ODD) superfamily (Lukačin et al., 2000; Kawai et al., 2014). F3H catalyzes the hydroxylation of (2S)-flavanones to dihydroflavonols, providing direct substrates for FLS. Then FLS desaturases the C-ring of dihydroflavonols to flavonols (**Figure 2A**). F3H exhibits a relatively narrow substrate specificity (Martens et al., 2003; Turnbull et al., 2004). While FLS displays some degree of promiscuity in the substrate preferences and catalytic activities. Several FLSs also possess F3H activity, such as CitFLS of citrus (*Citrus unshiu*), AtFLS1 of arabidopsis (*Arabidopsis thaliana*), and GbFLS of ginkgo (*Ginkgo biloba*), which can catalyze a series of reactions to produce flavonols with naringin as the substrate (Lukačin et al., 2003; Owens et al., 2008a; Xu et al., 2012). In this study, Two FLS genes, cloned from *R. chingii*, were demonstrated to have both hydroxylation and desaturation activities for flavanones. The catalytic activity of RcFLS1 was significantly higher than that of RcFLS2, suggesting that RcFLS1 might play a central role in flavonol synthesis in *R. chingii*. Sequence alignment revealed that RcFLS2 differs from RcFLS1 in several key amino acid residues, including changes in the characteristic “SxxTxLVP” motif (Serine replaced P228S, Proline at position 228) and several substrate binding residues (H128Y and E291V). This could be the direct cause of the low activity of RcFLS2. By aligning with other sequences of 2-ODDs involved in flavonoid biosynthesis, it was discovered that RcFLSs contain key residues described as being involved in F3H activity, which implies that RcFLSs have potential F3H catalytic activity. The kinetic analysis of enzyme activity indicated that the hydroxylation activity of RcFLS1 was significantly lower than that of desaturation activity. The tt6 (F3H-deficient) mutants can produce flavonols and PAs, but much less than the wild type (Peer et al., 2001; Owens et al., 2008b). Therefore, we speculate that RcFLSs mainly perform desaturation catalytic activity for dihydroflavonol, and only play a partially complementary F3H role in *R. chingii*.

### Competition between FLS and DFR can modulate the flavonoid metabolic flux in *R. chingii*

The flavonoid biosynthesis pathway in plants regards genetic and structural diversity, and dihydroflavonols are important intermediate substrates in the downstream pathway of flavonoid biosynthesis. The mechanism of the competition between FLS and DFR in the flavonoid biosynthesis pathway, which determines the final destination of metabolic flux, is unclear. The subcellular localization and interaction of these proteins showed a strong interaction between RcFLSs and RcDFR, which might form functional complexes in flavonoid metabolic processes. This study used the co-expression of RcFLSs and RcDFR in *E. coli* BL21 to compare the competitive relationship between RcFLSs and RcDFR. The result showed that the transgenic cell containing RcFLS1 and RcDFR genes mainly produced flavonols when flavanones (N and E) or dihydroflavonols (DHK and DHQ) were added as substrates. However, the cell containing RcFLS2 and RcDFR mainly accumulated leucoanthocyanidins feeding dihydroflavonols as substrates, possibly the reason for the very low catalytic activity of RcFLS2. Co-expressions of RcFLSs and RcDFR in tobacco, strawberry, and *Arabidopsis* furtherly verified the above results *in vivo*. We suggested that RcFLS1 was dominant in the competition with RcDFR, directing the intermediate substrate toward flavonols.

The low-catalytic activity or nonfunction *FLS* gene might have other roles in plant flavonoid metabolism. For example, three *FLS* genes were obtained in the genome of *Camellia sinensis*, while the catalytic activity of CsFLSs was significantly different (Jiang et al., 2020). The *CsFLSa*, the low catalytic capacity gene, has a higher expression level (ten times higher than other *CsFLS* genes) in young shoots of tea plants. Unlike *R. chingii*, tea leaves are rich in PAs, which may also be due to the competition between CsFLSa (divided into cluster II and RcFL2, **Figure S2**) and CsDFR. Recent research confirmed that the structural proteins in the flavonoid metabolic pathway form metabolon (weakly-bound multi-enzyme complexes of enzymes) to improve catalytic efficiency (Fujino et al., 2018; Nakayama et al., 2019). Combined with the above results, we speculated that FLSs might play a central role in assembling the flavonoid metabolon, which could switch on or off the flavonol metabolic flux by regulating active or inactive enzyme abundance. The competitive relation of FLS and DFR for controlling the metabolic flux should be further studied.

In addition, it has been suggested that flavonols (quercetin and myricetin) could inhibit the activity of DFR towards dihydroflavonols (Trabelsi et al., 2008; Aiguo et al., 2022); we confirmed that the accumulation of flavonols (kaempferol and quercetin) could inhibit the enzyme activity of RcDFR, which might strengthen the competitiveness of RcFLS1. It has been reported that the expression of *FLS* and *DFR* may be regulated by feedback mechanisms (Yin et al., 2012; Liu et al., 2013; Liao et al., 2015). Therefore, the regulation of metabolic flow in this way is not only controlled by the level of gene transcription but also depends mainly on the competition at the protein level.

**Figure 7:**
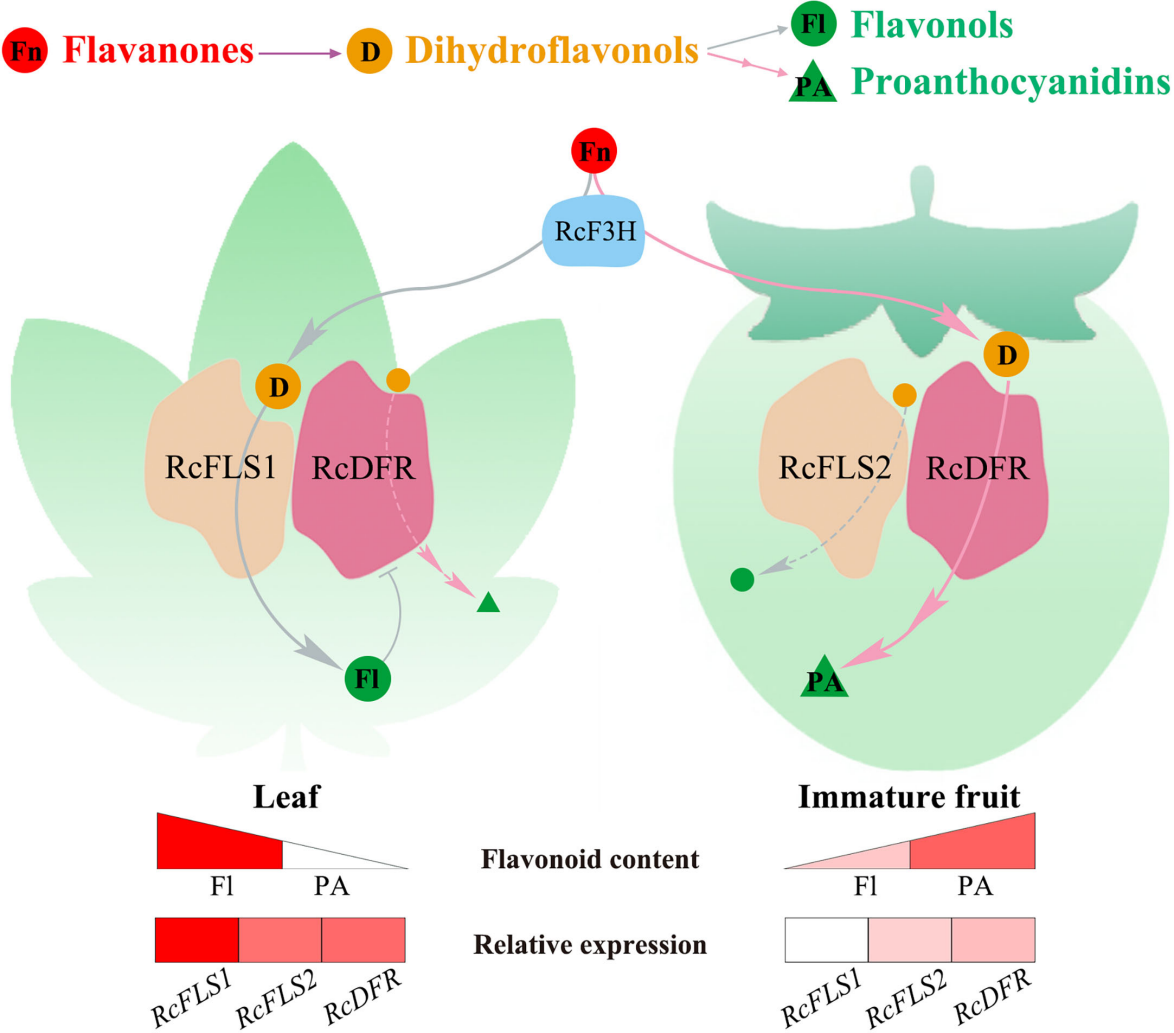
The model of the flavonoid metabolic flux in *R. chingii* leaves and fruits. Red spheres represent flavanones (Fn), orange spheres represent dihydroflavonols (D), green spheres represent flavonols (Fl), and green triangles represent proanthocyanidins (PA). The gray lines flow to flavonols, and the pink lines to proanthocyanidins. Spheres or triangles with letters indicate major metabolic flows. The triangular color blocks indicate the relative content of flavonoids, and the rectangular color blocks indicate the relative expression of *RcFLSs* and *RcDFR* genes.

The competition between FLS and DFR in *R. chingii* determines whether dihydroflavonols are converted to flavonols or PAs. We hypothesized that when RcFLS1 and RcDFR are both highly expressed in *R. chingii* (in leaves), they jointly perform catalytic functions in the metabolon, and metabolic flux is shifted to flavonols due to the stronger competitiveness of RcFLS1; when RcFLS1 is absent (in fruits), RcFLS2 and RcDFR are in the metabolon, and metabolic flux is shifted to PAs (**Figure 7**). This competition modulates metabolic flow at both the transcriptional level and enzymological perspective. *R. chingii* has a unique pattern of flavonoid synthesis that regulates the distribution of flavonols and PAs through competition between FLS and DFR, providing the metabolic basis for its unique health benefits.

## Data availability statement

Publicly available datasets were analyzed in this study. This data can be found here: https://www.rosaceae.org/Analysis/11326199.

## Author contributions

The presented study was conducted in collaboration with all authors. TL, LG, and YW designed the experiments. The experiments were performed by TL, JH, ZF, and CG. The data was analyzed by TL, HR, WQ, and NZ. TL, YL, and ZW wrote the manuscript. All authors contributed to the article and approved the submitted version. All authors contributed to the article and approved the submitted version.
