# Supplementary material for: Competition between FLS and DFR regulates the distribution of flavonols and proanthocyanidins in Rubus chingii Hu
Source: Front Plant Sci. 2023 Mar 8;14:1134993. doi: 10.3389/fpls.2023.1134993 (PMC10031046; doi:10.3389/fpls.2023.1134993)
Supplement: Supplementary file 1 [file DataSheet_1.pdf]

Table S1 Primers are used for gene cloning and expression.

Name	Sequence (5'-3')
RcFLS1-F	ATGGGGGTTGAGAGAGTGCAAGAC
RcFLS1-R	TTACTGGGGAATCTTGTTGAGCTTG
RcFLS2-F	ATGGAGGTAGTGAGAGTTCAAAC
RcFLS2-R	CTATTGTGGGATAGAGTTGAATTTG
RcDFR-F	ATGGGATCGGAGTCCGAATCCG
RcDFR-R	TTAGGCGGTGAGTTTGACATGGAC
pMAL-RcFLS1-F	ggaaggatttcagaattcggatcc ATGGGGGTTGAGAGAGTGCAAGAC
pMAL-RcFLS1-R	gtgccaagcttgcctgcaggtcgac TTACTGGGGAATCTTGTTGAGCTTG
pMAL-RcFLS2-F	ggaaggatttcagaattcggatcc ATGGAGGTAGTGAGAGTTCAAAC
pMAL-RcFLS2-R	gtgccaagcttgcctgcaggtcgac CTATTGTGGGATAGAGTTGAATTTG
pMAL-RcDFR-F	ggaaggatttcagaattcggatcc ATGGGATCGGAGTCCGAATCCG
pMAL-RcDFR-R	gtgccaagcttgcctgcaggtcgac TTAGGCGGTGAGTTTGACATGGAC
pRSF-RcFLS1-F(EcoRI)	caccacagccaggatccgaattcg ATGGGGGTTGAGAGAGTGCAAG
pRSF-RcFLS1-R(EcoRI)	gtcgacctgcaggcgcgccgagct CTGGGGAATCTTGTTGAGCTTG
pRSF-RcFLS2-F(EcoRI)	caccacagccaggatccgaattcg ATGGAGGTAGTGAGAGTTCAAAC
pRSF-RcFLS2-R(EcoRI)	gtcgacctgcaggcgcgccgagct TTGTGGGATAGAGTTGAATTTGC
pRSF-RcDFR-F(XhoI)	gatcgctgacgtcggtaccctcgag ATGGGATCGGAGTCCGAATCC
pRSF-RcDFR-R(XhoI)	cgcagcagcggtttctttaccaga GGCGGTGAGTTTGACATGGAC
cMYC-RcFLS1-F	ccggggatcctctagagtcgac ATGGGGGTTGAGAGAGTGCAAG
cMYC-RcFLS1-R	gctttgaatcgataccgactagt CTGGGGAATCTTGTTGAGCTTG
cMYC-RcFLS2-F	ccggggatcctctagagtcgac ATGGAGGTAGTGAGAGTTCAAAC
cMYC-RcFLS2-R	gctttgaatcgataccgactagt TTGTGGGATAGAGTTGAATTTGC
cFLAG-RcDFR-F	cagggtacccggggatcc ATGGGATCGGAGTCCGAATC
cFLAG-RcDFR-R	gaactagtgtcgactctaga GGCGGTGAGTTTGACATGGAC
pRSF-CsFLSa-F(EcoRI)	caccacagccaggatccgaattcg ATGGAGGTAGAGAGAGTGCAAG
pRSF-CsFLSa-R(EcoRI)	gtcgacctgcaggcgcgccgagct TTGGGGAATCTTATTGAATTTGC
pRSF-CsFLSb-F(EcoRI)	caccacagccaggatccgaattcg ATGGAGTTGGAGAGGGTGCAAG
pRSF-CsFLSb-R(EcoRI)	gtcgacctgcaggcgcgccgagct CTGGGGAAGCTTGTTCAGCTTAC
pRSF-CsDFR-F(XhoI)	gatcgctgacgtcggtaccctcgag ATGAAAGACTCTGTTGCTTCTGC
pRSF-CsDFR-R(XhoI)	cgcagcagcggtttctttaccaga AACCTTGTTGCCATTGACAGGG

Table S2 The relative content of flavonol derivatives in different tobacco leaves.

Compound	CK-1	CK-2	CK-3	RcFLS1-1	RcFLS1-2	RcFLS1-3	RcFLS2-1	RcFLS2-2	RcFLS2-3	RcDFR-1	RcDFR-2	RcDFR-3	RcFLS1+DFR-1	RcFLS1+DFR-2	RcFLS1+DFR-3	RcFLS2+DFR-1	RcFLS2+DFR-2	RcFLS2+DFR-3
**Co-injection (DHK)**																		
Kaempferol-3-O-glucoside	259	771	476	20509	41619	29937	1281	2841	2230	1109	1210	1124	31513	29491	30062	1212	749	1021
Kaempferol-3-coumaroylglucoside	1231	4215	3363	5282	15901	10830	3951	10306	7795	5221	7208	5827	21872	19826	20965	5530	3616	5051
Kaempferol-3-O-glucosylglucoside	85	171	204	7405	17440	13841	1319	2805	2215	329	479	472	7613	6965	7554	324	269	367
Kaempferol-3-O-rhamnosylglucoside-7-O-glucoside	710	1982	1728	4711	12930	9711	3480	10080	6843	2782	5845	4130	12047	9998	11437	3682	2316	3311
Quercetin hexose	961	2157	793	948	2983	1027	622	1124	332	4698	1075	630	5568	1396	1812	3265	619	779
Quercetin-3, 7-di-O-rhamnoside	6	14	9	6	11	11	3	15	10	16	23	24	15	20	17	13	11	12
Quercetin-3-O-rhamnosylglucoside	123	898	432	133	1178	325	132	375	184	2073	1109	496	2228	715	1173	1440	434	606
Quercetin-3-O-rhamnosylglucoside-7-O-glucoside	100	6	11	35	85	85	27	98	60	36	50	26	168	159	168	30	20	46
**Co-injection (DHQ)**																		
Kaempferol-3-O-glucoside	100	158	220	927	1024	941	94	57	56	75	113	90	806	889	803	146	115	120
Kaempferol-3-coumaroylglucoside	3199	5917	4186	12762	14185	12707	2572	3078	2109	1748	3280	2431	3688	3975	4075	2997	3201	2945
Kaempferol-3-O-glucosylglucoside	213	220	220	592	634	570	154	142	87	38	84	88	639	662	642	167	193	160
Kaempferol-3-O-rhamnosylglucoside-7-O-glucoside	4262	4966	5055	10081	11464	9235	3822	4333	2603	865	1991	1598	3869	4293	4033	2938	3471	2956
Quercetin hexose	335	875	484	38624	40205	35739	408	438	317	524	645	408	16004	20060	17575	1218	1374	1218
Quercetin-3, 7-di-O-rhamnoside	23	27	22	11	8	21	7	10	6	4	9	8	12	5	12	12	10	8
Quercetin-3-O-rhamnosylglucoside	107	4238	367	5491	7225	5521	122	431	126	107	478	192	1418	1712	1444	1301	226	421
Quercetin-3-O-rhamnosylglucoside-7-O-glucoside	40	105	95	5899	6470	5827	50	24	19	52	73	74	2202	2277	2006	188	220	259
