# Supplementary material for: Competition between FLS and DFR regulates the distribution of flavonols and proanthocyanidins in Rubus chingii Hu
Source: Front Plant Sci. 2023 Mar 8;14:1134993. doi: 10.3389/fpls.2023.1134993 (PMC10031046; doi:10.3389/fpls.2023.1134993)
Supplement: Supplementary file 2 [file DataSheet_2.pdf]

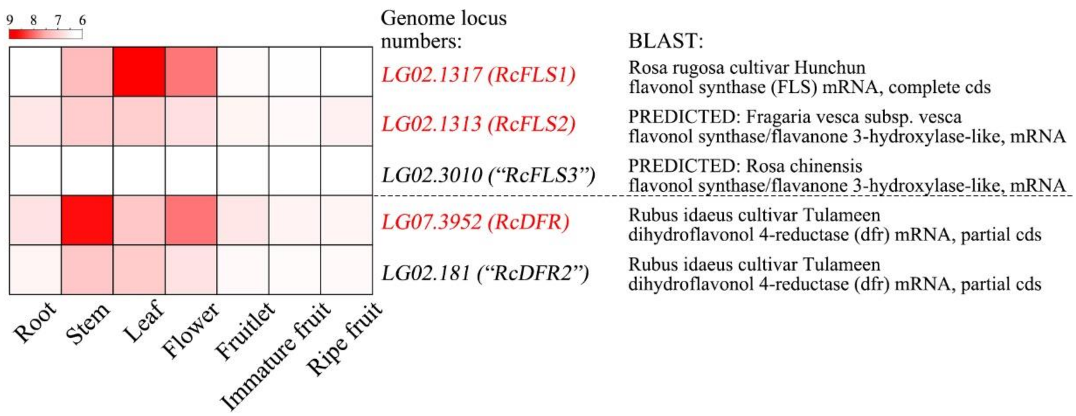

Figure S1. All homologs of *FLS* and *DFR* encoding genes in the *R. chingii* genome.

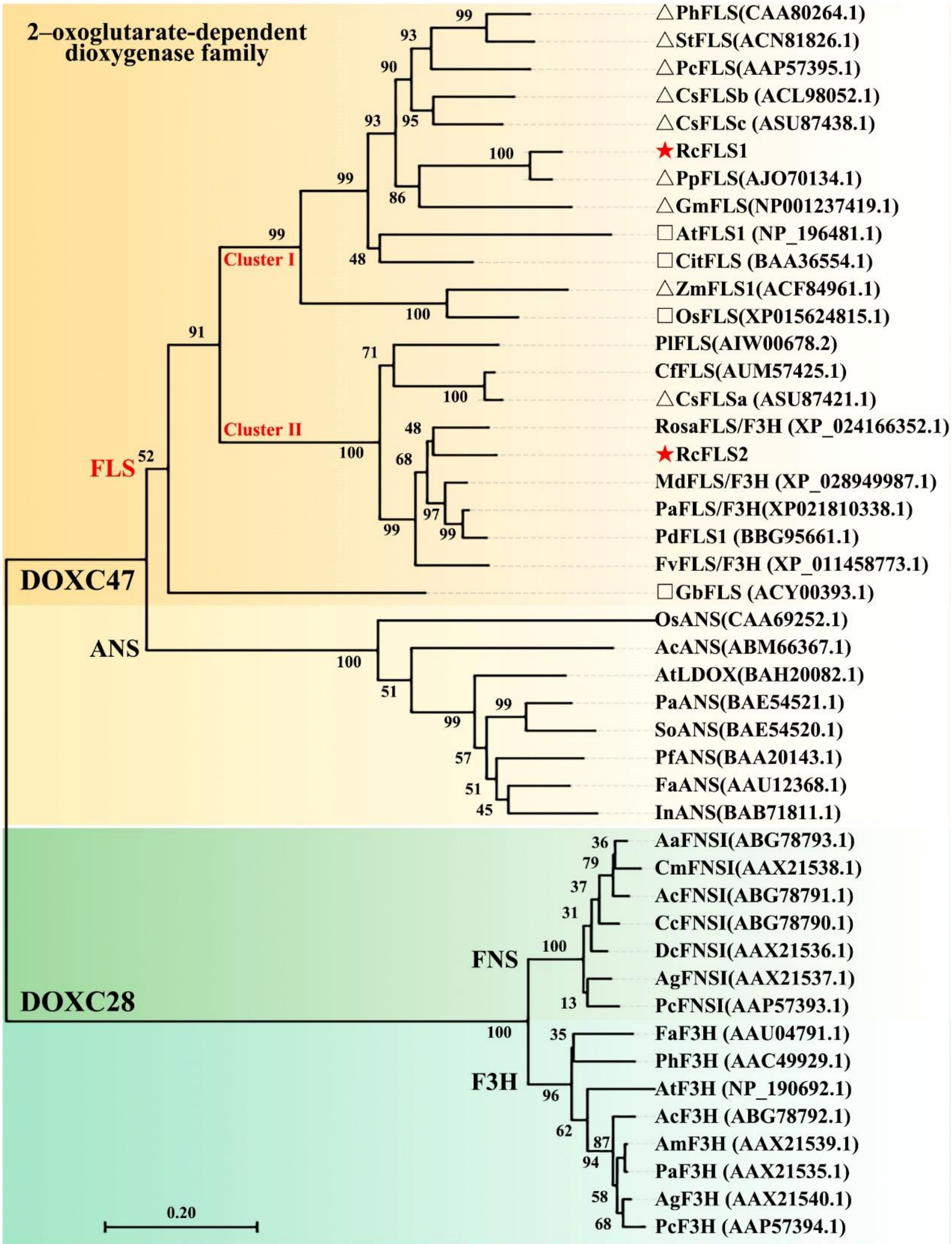

Figure S2. Phylogeny of flavonoid pathway 2-ODDs, including FLS, ANS, FNS, and F3H. The phylogenetic tree was constructed using the MEGA7 software with neighbor-joining method. The DOXC47 clade is on a yellow background, and the DOXC28 clade is on a green background. The FLS subclade is divided into two clusters, indicated in red font. Triangle-labeled FLS genes were reported to have flavonol synthase (FLS) activity, and square-labeled FLS genes were reported to have both flavonol synthase (FLS) and flavanone 3-hydroxylase (F3H) activities.

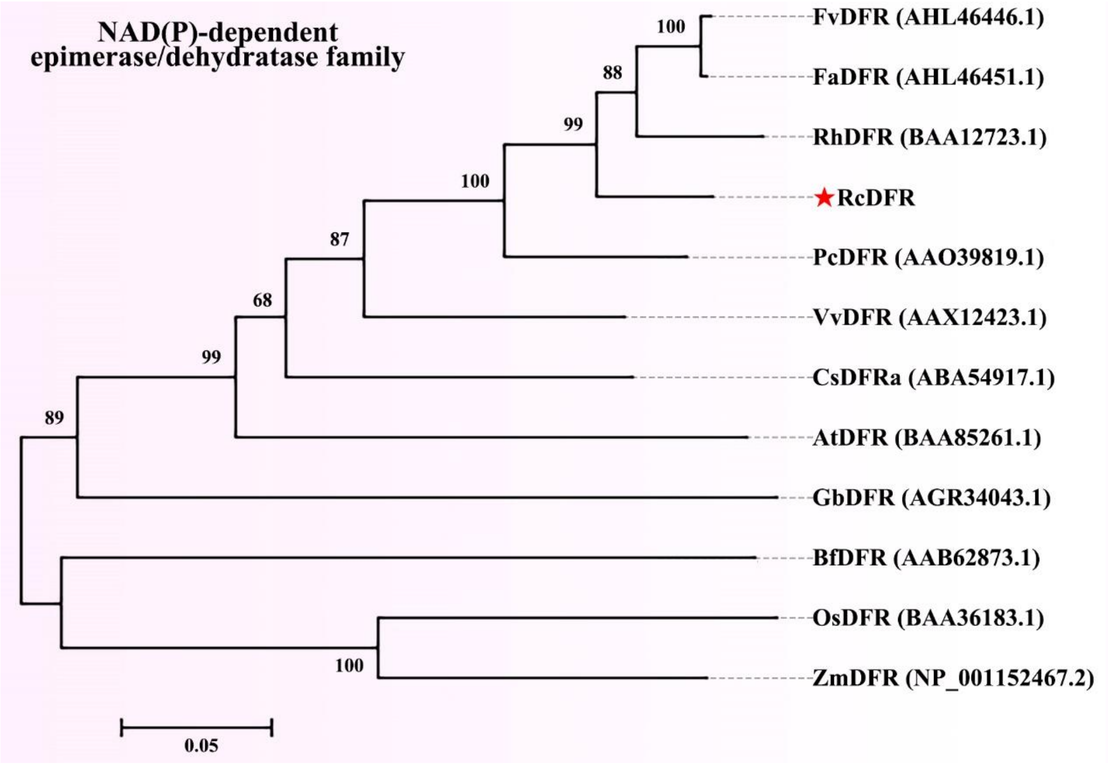

Figure S3. Phylogenetic tree of RcDFR and other DFRs from different plant species.

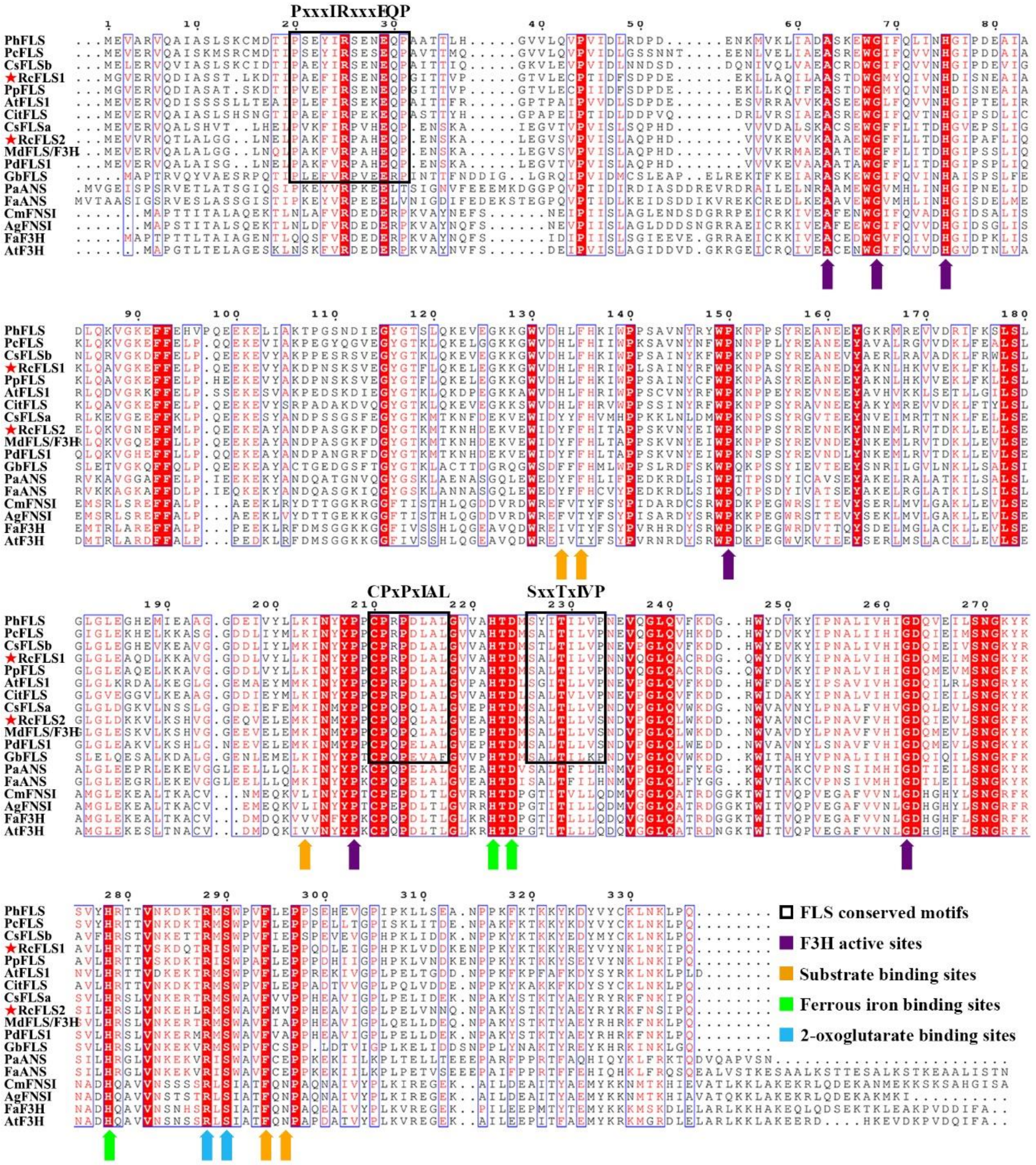

Figure S4. Multiple sequence alignment of RcFLSs with other 2-ODDs involved in the flavonoid biosynthetic pathway.

Multiple sequence alignments were performed using ClustalW. Conserved amino acids and motifs were labeled as follows: the FLS conserved motifs “PxxxIRxxxEQP,” “CPxPxLAL,” and “SxxTxLVP” are shown in BLACK, while residues involved in the substrate, ferrous iron, and 2-oxoglutarate binding are marked in ORANGE, GREEN, and BLUE, respectively. Residues relevant to F3H activity are shown in PURPLE.

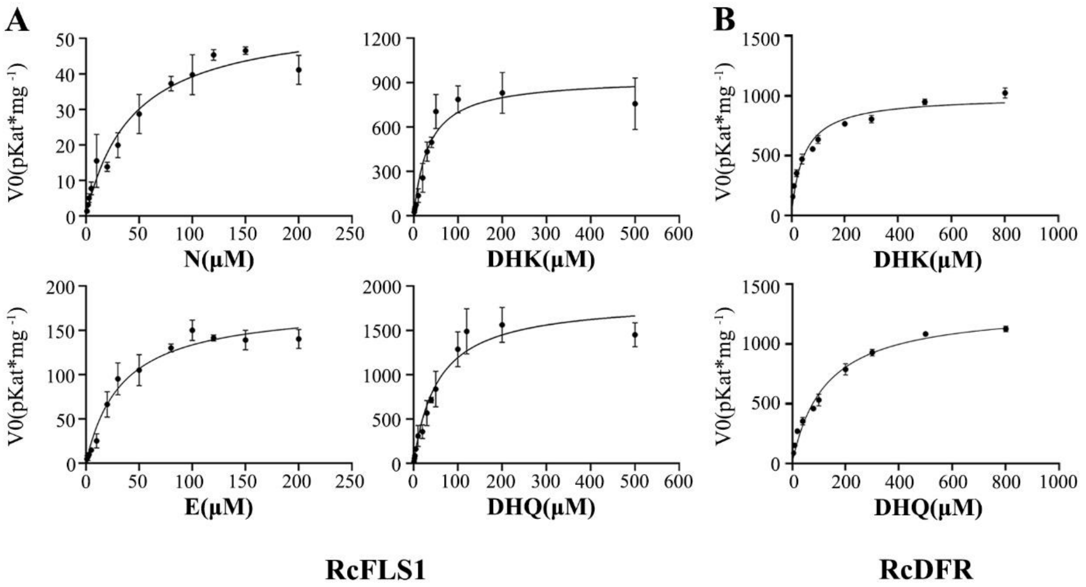

Figure S5. The Michaelis-Menten equation curve of recombinant RcFLS1 and RcDFR proteins.

The graph was plotted using Prism 8 software. The Michaelis-Menten constant was determined by averaging values from fitting three separate data sets.

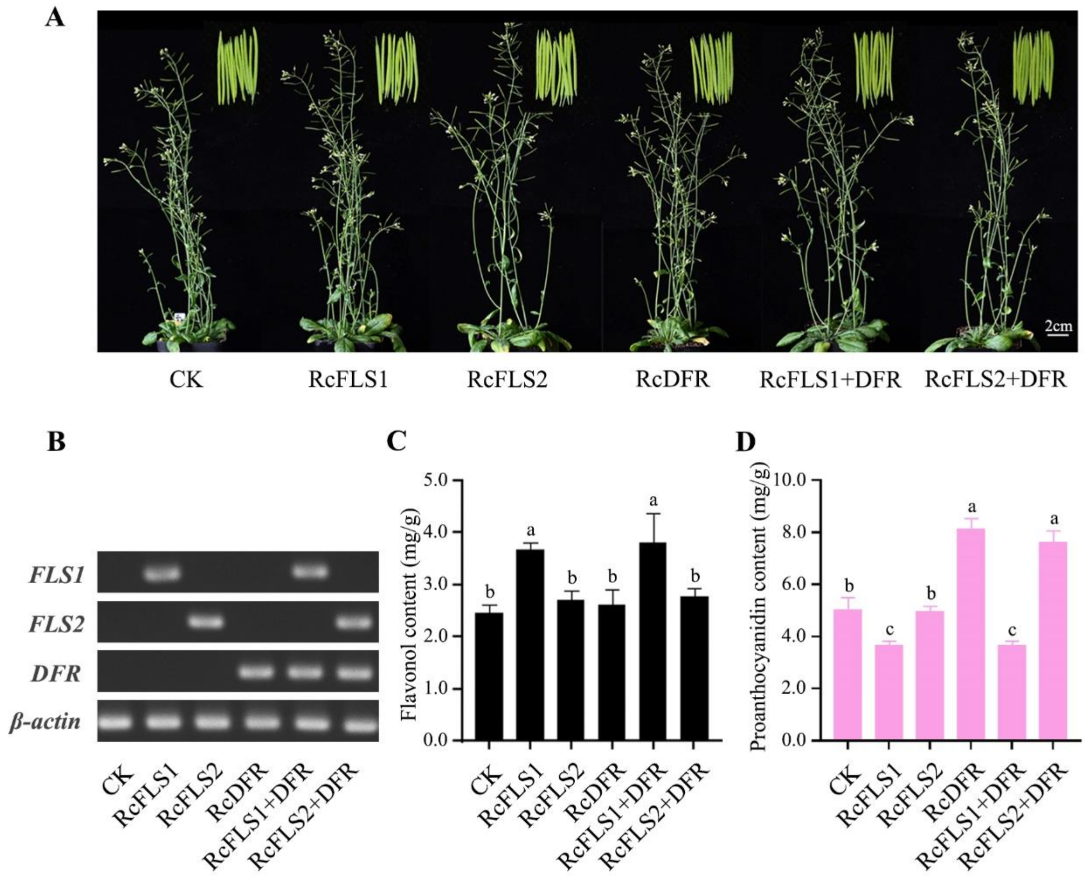

Figure S6. Assays of competition between RcFLSs and RcDFR in *Arabidopsis thaliana*.

(A) Phenotypes of transgenic *Arabidopsis* plants overexpressing *RcFLSs* and *RcDFR*. *Arabidopsis* green pods are shown in enlarged views. (B) Semiquantitative RT-PCR analysis of the transcription levels of *RcFLSs*, *RcDFR,* and housekeeping gene *β-actin*. (C) The content of flavonols extracted from transgenic *Arabidopsis* green pods. (D) The content of PA extracted from transgenic *Arabidopsis* green pods. Error bars indicate ± SD from three repeats. Statistical significance was determined by one-way ANOVA with post hoc Tukey's tests ($P < 0.05$) and indicated by letters.
